# A Qualitative Content Analysis of Perceived Individual and Relational Consequences of Sexual Compliance and Their Contributors

**DOI:** 10.1007/s10508-024-02948-9

**Published:** 2024-07-18

**Authors:** Annika Gunst, Katarina Alanko, Sabina Nickull, Marieke Dewitte, Marianne Källström, Jan Antfolk, Patrick Jern

**Affiliations:** 1 Department of Psychology, Åbo Akademi University, Tehtaankatu 2, 20500 Turku, Finland; 2 Department of Clinical Psychological Science, Maastricht University, Maastricht, The Netherlands

**Keywords:** Sexual compliance, Sexual desire, Unwanted sex

## Abstract

Sexual compliance (i.e., consensually engaging in sex despite a lack of desire for it) is common in committed intimate relationships, but the consequences of compliance for the well-being of the individual and the relationship are poorly understood. We investigated the perceived consequences of sexual compliance and perceptions of factors contributing to negative/positive consequences by applying qualitative content analysis to free-text retrospective survey responses from 107 (mostly) Finnish adults. We identified five themes of personal consequences (emotions and mood, sexual experience, sexual desire, pressure and violations, and physical pain), four of relational consequences (relationship satisfaction, partner’s response, relationship interaction, and value alignment), and nine of possible factors contributing to negative/positive consequences (communication, self-esteem, motives for sex, relationship factors, agency and self-knowledge, mental health and stress, psychological flexibility, societal norms, and past negative experiences). Perceived consequences varied widely across individuals, both in terms of whether any positive or negative consequences were experienced and whether compliance was perceived as improving or worsening specific domains of well-being. We discuss the themes identified in relation to previous theories of sexuality and intimate relationships and offer hypotheses that can be tested in future quantitative studies.

**Supplementary Information:**

The online version contains supplementary material available at 10.1007/s10508-024-02948-9.

## Introduction

Sexual desire is commonly defined as an interest in or personal motivation to engage in sexual activity (e.g., Regan, 1999). Concerns about sexual desire are among the most commonly reported sexual complaints and can have negative consequences for the individual and the relationship (e.g., Dewitte et al., 2020; Gunst, 2019). Individuals in committed, intimate relationships often face the issue of sexual desire discrepancy, situations in which the desire for sex does not match between partners (Day et al., 2015). Such situations can lead to *sexual compliance*, that is, consensually engaging in partnered sexual activity despite a lack of desire for it (e.g., Impett & Peplau, 2003; Katz & Tirone, 2009). Conceptually, sexual compliance differs from sexual coercion and assault in that it involves consent as well as the absence of physical and psychological pressure and coercion by the partner.

Previous research in Western populations has shown that sexual compliance is common in committed intimate relationships (e.g., Himanen & Gunst, 2023; Vannier & O’Sullivan, 2010; Willis et al., 2022). While most existing research has focused on sexual compliance among cis women in heterosexual relationships, sexual compliance is not limited to this population (Himanen & Gunst, 2023; Khera et al., 2022; Quinn-Nilas et al., 2013; Rubinsky, 2020). Previous research suggests that compliance may have both negative and positive consequences for the well-being of the individual and the relationship. However, these studies are fragmentary in terms of the consequences included, and few have examined when and how sexual compliance might be beneficial or harmful. In a recent position statement, the European Society for Sexual Medicine called for more research to support the development of clinical guidelines for issues related to desire discrepancy (Dewitte et al., 2020).

### Consequences of Sexual Compliance

In relationship research, interdependence theory suggests that people may choose to transform their own motives for the sake of their partners’ desires in order to achieve mutually beneficial relationship outcomes (Kelley, 2013). Such sexual transformations are generally viewed as part of a relationship maintenance strategy. For example, one study found that one of the most common self-reported strategies women used to cope with desire discrepancy was to comply with sex and try to meet their partner’s needs (Herbenick et al., 2014). A couple of studies in Western populations indicate that there are positive relationship outcomes of sexual compliance. In an older survey study of 160 university students followed over a 2-week period, the participants reported several perceived positive relationship outcomes of compliance: the partner’s satisfaction, promoting intimacy, and preventing disagreements in the relationship (O’Sullivan & Allgeier, 1998). In a cross-sectional study using online survey data from 1496 Finnish individuals currently or recently in a committed relationship, the most commonly perceived positive consequences were increased intimacy and feelings of love and attachment (Himanen & Gunst, 2023).

In addition to these suggested positive relationship outcomes, individuals in long-term relationships often report that their desire is “responsive” (i.e., emerges after sexual activity has already begun; Basson, 2000). Psychoeducation about this responsive quality of desire—that sexual activity can trigger sexual desire—is commonly included when working with concerns related to sexual desire (e.g., Nagoski, 2015), thereby promoting engagement in sex without strong initial interest.

On the other hand, studies also suggest that sexual compliance may have negative consequences for the individual and the relationship. For instance, in a study analyzing survey data and saliva samples from 64 university students in monogamous romantic relationships, individuals who complied more frequently had higher cortisol levels, an indicator of stress (note, however, that this might reflect a general predisposition to higher stress levels in these individuals; Hartmann & Crockett, 2016). In a study using daily diary data collected over 3 weeks from 63 young adults in heterosexual relationships, the participants rated sexually compliant events ass less enjoyable compared to desired sexual activity (Vannier & O’Sullivan, 2010). O’Sullivan and Allgeier (1998) found that among those who reported perceived negative consequences of sexual compliance, the most common consequences were emotional discomfort and feeling disappointed in themselves. In a cross-sectional online survey including data from 530 individuals in non-normative relationships (e.g., LGBT+, polyamorous, and BDSM), those who complied reported poorer mental health and lower relationship satisfaction compared to those who did not comply (Rubinsky, 2020). Interviewing 41 young US women, Bay-Cheng and Bruns (2016) found some perceived negative consequences for self-perception and future intimate relationships. Himanen and Gunst (2023) found that the most commonly reported negative perceived consequences were decreased mood, self-esteem, relationship satisfaction, and sexual satisfaction.

### Factors Contributing to Positive and Negative Consequences

There is reason to believe that the outcome of sexual compliance varies based on individual and relational factors. For instance, in a correlational survey study, Katz and Tirone (2009) examined 193 US female university students in heterosexual relationships. They found that avoidance motives (i.e., having sex to avoid the negative outcome of declining) were associated with lower relationship satisfaction, whereas approach motives (i.e., having sex to achieve a positive outcome) were not. Women who complied frequently and reported high levels of avoidance motives reported the lowest relationship satisfaction. In support of this, Himanen and Gunst (2023) also found that avoidance motives were associated with reporting more perceived negative consequences of compliance.

Studies have also shown that individuals who report more positive feelings about making sexual transformations for the sake of their partner’s desires report greater sexual relationship satisfaction than individuals who report fewer positive feelings about making such transformations (Burke & Young, 2012). Muise and Impett (2015) found that individuals who were highly motivated to make sexual transformations, or what they termed high in sexual communal motivation, were more satisfied with and committed to their relationships.

While sexual compliance is not limited to heterosexual relationships, power dynamics and sexual decision-making within these relationships, often informed by gender norms and structures, can shed light on broader patterns of compliance. For example, Conroy et al. (2015) argued that while sexual compliance by definition excludes partner pressure and manipulation, it may include other, more covert aspects of coercion stemming from societal norms and gendered power structures in intimate relationships. Gavey (1992) called such discourses technologies of heterosexual coercion. In French and Neville’s (2017) qualitative study of 25 adolescent US women, many participants described young women feeling pressure to comply with sexual advances to please a male partner. Farvid and Saing (2022) further challenged the view of sexual compliance as consensual sex, arguing that consent is constrained in situations of compliance. In their qualitative study of 11 married Cambodian women, participants often complied with sex in order to avoid negative consequences if they refused. Relating to power dynamics in intimate relationships outside a gendered context, Himanen and Gunst (2023) found that individuals who experienced less authority in their sexual relationship than their partner also reported more perceived negative consequences of compliance. Bay-Cheng and Bruns (2016) found that the perceived consequences varied by socioeconomic status. While most participants with a higher socioeconomic status reported either no perceived consequences or positive ones, most participants with a lower socioeconomic status reported perceived negative consequences of compliance. Given these more covert forms of pressure, it is perhaps unsurprising that sexual compliance can adversely affect mental and relational well-being.

Taken together, the literature suggests that compliance has both negative and positive consequences for the well-being of the individual and the relationship, and a few studies suggest possible factors contributing to negative/positive consequences. However, previous studies have often relied on predetermined variables that constrain the scope of inquiry. To achieve a more comprehensive understanding of individual experiences of the consequences, the current study employed an inductive qualitative approach, allowing for an in-depth examination of the complexities that quantitative methods might not provide.

### Aims and Research Questions

In the present study, we aimed to qualitatively investigate the perceived consequences of sexual compliance and the perceptions of factors that contribute to negative and positive consequences. We wanted to increase the knowledge about when and how sexual compliance may benefit or harm the well-being of the individual and the relationship. We used open-ended questions to identify potentially relevant areas that the previous studies may have overlooked. Our study had six specific research questions:

1. What are the perceived (a) negative and (b) positive consequences of sexual compliance (c) for the individual and (d) for the relationship?
2. What personal and relational factors are perceived to contribute to the (a) negative and (b) positive consequences of sexual compliance?

## Method

### Participants and Procedure

We created the online survey using a secure online survey platform. We collected data between May 4 and July 25, 2022. The survey was available in English, Finnish, and Swedish. We recruited participants through Facebook advertisements targeting individuals aged 18–65 in Finland. We also shared the survey on our research group’s webpage on the host university’s official website, and by sharing the webpage on our research group’s Facebook page and through our personal Facebook profiles. To obtain a more demographically diverse sample, the study was also shared in a Finnish Facebook group for men (*Miestenhuone*, “Men’s Room,” approximately 10,000 members) and a Finnish Facebook group for LGBTQ+ individuals (approximately 8900 members).

Before being able to participate, interested participants gave informed consent and assured that they met the inclusion criteria. Our inclusion criteria were (1) being at least 18 years old and (2) having complied to sexual activity in a committed relationship at least once. We described the anonymous and voluntary nature of the study, that anonymous data could be made openly available online, and that participants could discontinue their participation at any time, but that data already collected could not be removed. We also provided definitions for key aspects of the study in the beginning of the survey. Sexual compliance was defined as consensually engaging in sexual activity with a partner despite the lack (at least in the beginning) of sexual desire. Sexual activity was defined as a broad range of sexual behaviors that could include, for instance, touching/petting, oral sex, penetrative sex, or video/phone sex. Sexual desire was defined as being interested in and personally motivated to engage in sexual activity, with or without physical reactions (e.g., erection, tingling, and lubrication). We emphasized that the survey would ask the participants to describe their experiences of sexual compliance in their own words. Finally, we mentioned that participants who completed the survey would have the opportunity to enter a drawing for two 50€ gift cards to an online multi-brand clothing store.

After we distributed the survey, 282 individuals started it. Of these, 14 dropped out after giving informed consent, and another 62 dropped out before the free-text responses relevant to the current study. We also removed 21 responses that appeared to have been filled out by a bot (free-text responses were in Chinese, did not make sense, and/or were identical across several participants, and Likert responses were not logical). This left us with 185 participants who perceived any negative/positive consequences. Due to the nature of the participant recruitment, it was not possible to calculate a response rate.

Detailed frequencies of these individuals’ perceived consequences are presented in Online Supplementary Table 1. Of these 185 participants, 107 provided at least one free-text response and were included in the qualitative content analysis. Their demographic characteristics are presented in Table 1.

**Table 1:** Demographic characteristics of participants who provided at least one free-text response (*n* = 107)

Variable	*n*	%
Gender		
Woman	83	77.6
Man	20	18.7
Transwoman	0	0.0
Transman	1	0.9
Non-binary	3	2.8
Sexual orientation		
Heterosexual	73	68.2
Bisexual	22	20.6
Gay/lesbian	6	5.6
Pansexual	5	4.7
Asexual	1	0.9
Relationship status		
Single	36	33.6
In a dating relationship	4	3.7
In a committed relationship	20	18.7
In a consensual non-monogamous relationship	3	2.8
Cohabiting	21	19.6
Married	22	20.6
Other	1	0.9
Education		
Middle/junior high school diploma (15 years)	3	2.8
Vocational school or high school diploma (18 years)	21	19.6
Bachelor’s degree (applied or university)	37	34.6
Master’s degree	44	41.1
Licentiate/doctorate degree	2	1.9
Other	0	0.0
Occupation		
Studying	29	27.1
Employed or self-employed	69	64.5
Retired	1	0.9
Unemployed	2	1.9
Other	6	5.6
Monthly gross income		
Less than 500€	11	10.3
500–999€	13	12.1
1000–1999€	18	16.8
2000–2999€	29	27.1
3000–3999€	21	19.6
4000–4999€	9	8.4
5000–5999€	2	1.9
6000€ or more	4	3.7
Nationality		
Finnish	96	89.7
West European	3	2.8
North American	8	7.5
Survey language		
Finnish	74	69.2
Swedish	20	18.7
English	13	12.1

Mean age = 31.5 years (SD = 8.8, range 18–60). Mean relationship duration for those in relationships (*n* = 64) = 7.5 years (SD = 5.9, range 1.2–30.8)

A numerical breakdown of those who perceived any negative/positive consequences and those who provided free-text responses is presented in Table 2.

**Table 2:** Numerical breakdown of participants who perceived negative and/or positive consequences and provided free-text responses

	Perceived consequence		Free-text response	
	*n*	%	*n*	%
Personal				
Negative	104	56.2	83	79.8
Positive	90	48.6	78	86.7
Relational				
Negative	99	53.5	77	77.8
Positive	98	53.0	81	82.7
Contributing factors				
Negative	132	71.4	83	62.9
Positive	125	67.6	76	60.8

For contributing factors, responses were not provided separately for personal and relational consequences. The percentages for perceived consequences are based on those completing the questionnaire (*n* = 185). The percentages for free-text responses are based on those experiencing said consequence. Free-text response numbers represent responses after recoding misplaced responses (described in the Analytical Process section)

For comparison, the demographic characteristics of participants, including those who perceived no negative or positive consequences and those who perceived but did not provide any free-text responses (*n* = 185), are presented in Online Supplementary Table 2.

### Measures

Before the open-ended questions were administered, participants answered the following branching questions: “Has complying to sexual activities affected yourself personally (apart from any consequences it may have had on the relationship)?” and “Has complying to sexual activities affected the relationship?” with the response options (a) No; (b) Yes, it has had only negative consequences; (c) Yes, it has had only positive consequences; and (d) Yes, it has had both negative and positive consequences. Based on these responses, the participants were then asked to write down all negative and positive consequences for themselves personally and the relationship. Participants were further asked to identify any personal or relational factors they felt had contributed to the consequences by answering the following questions: “What personal factors (e.g., low self-confidence) or relationship-related factors (e.g., poor communication) do you think have contributed to the negative consequences of compliance?” and “What personal factors (e.g., high self-confidence) or relationship-related factors (e.g., good communication) do you think have contributed to the positive consequences of compliance?” Participants with negative and positive consequences for themselves and the relationship responded to all six questions (negative personal, positive personal, negative relationship, positive relationship, factors contributing to negative consequences, and factors contributing to positive consequences). Participants who did not perceive any consequences skipped the questions.

### Analytical Process

We analyzed the data using qualitative content analysis, a descriptive-interpretive qualitative method commonly used in psychological research (e.g., Mayring, 2000; Elo & Kyngäs, 2008). Our study had a predominantly inductive (rather than deductive) approach; we analyzed the data without any predetermined codes based on a specific conceptual framework. We did this because our study aimed to gain a comprehensive understanding of experiences of sexual compliance, allowing for experiences that have not been previously examined in the literature. In addition, our study took an experiential (rather than critical) approach; we valued respondents’ experiences as personal states held by themselves rather than making claims about the social constructions behind the participants’ expressions. We did this because we were primarily interested in the participants’ subjective experiences of sexual compliance and not the social constructs behind their responses. Finally, our study combined a semantic and latent approach; that is, we constructed codes both on the basis of their surface meaning and, in cases where we found it relevant, we attempted to identify underlying meanings or assumptions.

We followed the analytical steps of qualitative content analysis (preparation, organization, and presentation; Elo & Kyngäs, 2008) separately for our six research questions. The first author, a female-identifying licensed clinical psychologist, authorized sex counselor (Basic Sexology), and postdoctoral researcher with a background in sex research, acted as the main coder. First, the main coder read through the data several times to get a better sense of the material. Second, the main coder created initial codes, reviewed and compared them, and grouped them into higher-order, specific categories and, finally, more overarching themes. To promote consistency in the coding, a cyclical approach was used, in which the data were reviewed in multiple iterations. On two occasions during the process, one female and one male co-author acted as co-coders in a more reflexive manner by sense-checking ideas and reviewing uncertain interpretations.

Coding was done by writing comments next to the text in MS Word. Some single codes (e.g., *dirty sheets* and *liberated sex life*) did not fit into any overarching theme and were omitted. The number of omitted codes for each research question ranged from 7 to 18. In some cases, the participants had provided relational consequences among the free-text responses for personal consequences and vice versa. These consequences were coded and moved (e.g., the code *less trust in partner* was moved from personal to relational consequences, and the code *decreased self-esteem* was moved from relational to personal consequences). A few individuals who had reported experiencing no, for instance, personal consequences, still provided examples of personal consequences in the free-text answer for relational consequences. These responses were treated as if the participant had experienced personal consequences, adding six individuals to the negative personal consequences, two individuals to the negative relational consequences, 14 individuals to the positive personal consequences, and two individuals to the positive relational consequences. In addition, one individual whose only response among the negative relational consequences was treated as a personal consequence was removed from the frequency of individuals with negative relational consequences. The specific frequencies of individuals reporting negative/positive personal/relational consequences are presented in Table 2.

When the coding was finished, one female and one male co-author coded approximately 30% of the responses for each of the six research questions to assess the rigor and transparency of the coding and to ensure communicability across individuals. The co-authors were given a list of the specific categories with a few examples of each category. Cases where both co-coders had chosen a different category, and where both did not choose any category, were reviewed by the main coder, and the rest of the responses were checked for similar cases. An inter-rater reliability estimate was then calculated by dividing the number of agreements by the number of agreements plus the number of disagreements (including new suggested codes). This yielded a sufficient reliability estimate of 80.52% (Miles & Huberman, 1994).

Finally, we prepared a report of the analysis, including frequency calculations for each detailed category. Because we wanted to provide an illustrative and descriptive account of the analysis, we kept most of the interpretive presentation in the discussion section. This also allowed us to discuss conceptually separate categories together when relevant (e.g., when discussing themes present in the previous literature). For the frequency reports, duplicates within the detailed categories (i.e., cases where an individual provided a response that resulted in two or more codes ending up in the same category) were marked, and unique frequencies (i.e., unique individuals reporting a particular category) and total frequencies (i.e., the total number of codes for a detailed category, including duplicates) were calculated. Finally, we examined whether the detailed categories were reported across genders and among both the 30 youngest (ages 18–26) and 30 oldest (ages 35–60) participants who provided at least one free-text response.

## Results

The analysis yielded five overarching themes for personal consequences, four for relational consequences, and nine for factors contributing to the consequences. Table 3 provides a summary of the overarching themes. A summary of the more detailed categories (i.e., subcategories of the overarching themes), their frequencies, and information regarding whether the category was reported by both cis women and cis men is presented in Online Supplementary Table 3.

**Table 3:** Summary of the overarching themes for perceived consequences and their predictors

Research question	Theme	Negative	Positive
Personal consequences	Emotions and mood	x	x
	Sexual experience	x	x
	Sexual desire	x	x
	Pressure and violations	x	
	Physical pain	x	
Relational consequences	Relationship satisfaction	x	x
	Value alignment		x
	Partner’s response	x	x
	Relationship interaction	x	x
Factors contributing to negative/positive consequences	Communication	x	x
	Self-esteem	x	
	Motives for sex	x	x
	Agency and self-knowledge	x	x
	Relationship factors	x	x
	Mental health and stress	x	
	Societal norms	x	
	Psychological flexibility		x
	Negative experiences	x	

The Negative and Positive columns indicate whether the theme was described as a negative and/or positive consequence, and whether it was proposed as a factor contributing to the negative and/or positive consequences. For instance, the theme Emotions and mood included both negative (i.e., participants reporting negative emotions) and positive (i.e., participants reporting positive emotions) consequences

Below, we present the overarching themes (subheadings) and give illustrative free-text examples of the detailed categories. We provide the best approximate translation for responses in Finnish and Swedish (our research team included Finnish and Swedish native speakers). To avoid the potential identification of participants with less common identity labels, especially in cases where it is known that a particular individual participated in our study, age is shown as an aggregate, and individuals with at least one identity label with three or fewer respondents (transman, non-binary, asexual, and consensual non-monogamous relationship) were labeled as “less represented identity participants.” The percentages illustrate the number of individuals in a specific detailed category (e.g., negative feelings about self) of those who provided a response to the specific research question (e.g., negative personal consequences). A longer list of illustrative extracts is presented in Online Supplementary Table 4.

The length and level of detail in the responses varied greatly. Some responses consisted of only one or a few words (e.g., “anxiety”), whereas other participants gave more elaborate descriptions of their experiences. The examples below represent some of the more detailed responses to give a more thorough description of the participants’ experiences. Some of the examples presented below were included in several themes and/or detailed categories (e.g., “Caused my partner to later rape me” was coded as both problematic future sexual dynamic and feeling violated/pressured). Note that we did not include illustrative examples from cis men for all detailed categories where there were responses from cis men. This decision was made because some of these responses were very brief, consisting of only one or a few words. While these short responses are representative of the categories, they do not add much value as examples. A breakdown of the detailed categories with responses from cis men is found in Online Supplementary Table 3.

For most themes, some individuals found that complying had a negative consequence, whereas others found that it had a positive one. In the following, we present the themes once, without dividing them into separate negative and positive themes.

### Personal Consequences of Compliance

The personal consequences were categorized into five overarching themes: emotions and mood, sexual experience, sexual desire, pressure and violations, and physical pain.

#### Emotions and Mood

The first overarching theme concerned the emotions and mood of the respondent. Forty-eight percent of those who perceived negative personal consequences reported negative feelings more generally. These feelings included, for instance, anxiety, regret, frustration, sadness, emptiness, and disgust. The participants reported both negative feelings at the event of compliance and long-term negative feelings due to compliance. For this theme, several participants wrote only keywords (e.g., “anxiety” and “regret”) without expanding on the topic. In some cases, however, the participants described these feelings, emerging both during and after the event, in more detail:

Negative consequences have included, for example, anxiety that has risen during the situation. In the situation, it has still felt like I just have to get over the anxiety. I have also felt anxious afterward, resulting in tearfulness. (Heterosexual single woman, 25–34)

On the other hand, 27% reported that compliance had led to an improved mood or them feeling good:

Made me happy in the moment. (Less represented identity participant, 18–24)

Thirty-four percent reported negative feelings specifically about themselves. These included, for instance, feeling guilty, shame, inadequate, dirty, as well as loss of self-esteem and self-worth. Again, several participants wrote only keywords, while a few expanded on the topic. While some participants perceived that compliance made them feel worse about themselves, 10% reported that it made them feel better. Lastly, 8% reported that compliance had led to negative feelings or thoughts about the future. Some participants worried about future interactions with their partners:

Compliance also increased the fear that this was to be expected in the future and contributed to anxiety when going to bed, knowing that the other person would be interested in having sex and I might not be. (Heterosexual single woman, 25–34)

Some participants, again, reported more long-term worry and hopelessness about future sex and relationships:

I am scared to enter another relationship because of the obligations I feel to have sex with that person and the fear of enduring frequent sexual compliance again. (Heterosexual single woman, 18–24)

#### Sexual Experience

The second overarching theme concerned consequences that were related to the compliant experience or activity in itself. Forty-one percent of the participants who reported any positive personal consequences mentioned that the sex—at least sometimes—turned out to be good or pleasurable:

Sexual pleasure could sometimes make the interaction worth it. (Heterosexual single man, 25–34)

On the other hand, 22% reported a decreased quality of sex. Some reports concerned a decreased amount of pleasure while complying:

I switch off completely and don’t feel pleasure in my body; for instance, when my partner wants to please me and gives me oral sex, I’m just bored and pretend to enjoy it. (Bisexual single woman, 25–34)

Other reports concerned, for instance, performance and routine and issues with relaxation and arousal. Twenty-one percent reported that they personally enjoyed or felt good about making their partner happy or satisfied:

It feels good to consider the other [person]. (Bisexual man in relationship, 25–34)

Eighteen percent reported that compliance had contributed to new experiences. Some mentioned that complying had changed how they viewed their own sexuality or introduced them to new activities that they enjoyed:

I’ve found new things that arouse me, although I was a bit skeptical at first (okay then, let’s try it if you want). (Bisexual cohabiting woman, 25–34)

Lastly, 6% reported that compliance meant wasting precious time or that other things were left undone:

I don’t just let myself be or rest, even if it would be beneficial for me. I may be tired, especially in the evenings when I have sex, or I may not have slept enough in the morning because I had sex. (Bisexual woman in relationship, 25–34)

#### Sexual Desire

The third overarching theme concerned sexual desire. Thirty-five percent reported that they experienced desire after starting the sexual activity:

[Compliance] can help to awaken my own desire; I have always ended up enjoying sex—a bit like testing the ice with a stick to see if the desire will ignite. (Heterosexual cohabiting woman, 25–34)

Five percent also reported that their desire had increased because of compliance. On the other hand, 18% reported decreased sexual desire or interest because of compliance. In some instances, the time frame of the decrease/increase was not clear, whereas in others, the decrease/increase was described as a long-term consequence:

An increase in my own sexual desire in the moment and also afterward. A couple of consecutive times [it] has raised my low desire for a longer period of time. (Heterosexual cohabiting man, 25–34)

#### Pressure and Violations

Even though sexual compliance per definition excludes situations where the individual is pressured, 24% of those who perceived negative personal consequences reported feeling pressured or violated. Some keywords used were degraded, disrespected, intruded, coerced, and used. The pressure was often described as covert; that is, the participant either did not mention explicitly that the partner put pressure on them or mentioned that the pressure came from society at large:

I have felt obligated to say yes and felt guilty if I refused completely. (Heterosexual married man, 25–34)

The societal pressure to be sexy in a relationship is ever-present. (Less represented identity participant, 25–34)

In some instances, however, it seemed like the feeling of pressure stemmed from the negative consequences that *not* complying might bring:

Sometimes I feel pressure to pretend to be more interested than I am at the moment so as not to offend my partner. (Bisexual woman in relationship, 25–34)

Twelve percent reported consequences related to their personal boundaries.

Being flexible feels difficult. Am I overstepping my boundaries? Am I allowed to just not want to? Do I need to right now? (Bisexual man in relationship, 25–34)

#### Physical Pain

Lastly, 21% reported physical pain. This pain was often reported together with insufficient arousal or relaxation:

Sex causes more pain if I am not properly aroused. Sometimes there may be pre-existing sores or other pain or irritation in the genitals that are forgotten when I am aroused but return worse after sex. (Bisexual married woman, 35–44)

One participant also reported vaginismus (i.e., involuntary vaginal muscle tension or spasms), which is often associated with discomfort and pain, especially during penetration, creating a vicious cycle of pain and muscle tension.

### Relational Consequences of Compliance

The relational consequences were categorized into four overarching themes: relationship satisfaction, partner’s response, relationship interaction, and value alignment.

#### Relationship Satisfaction

Forty-eight percent of the participants who reported any positive personal consequences mentioned that compliance had contributed to increased intimacy with their partner.

The sexual connection is maintained and sometimes strengthened, positive sexual experiences reinforce a sense of intimacy and belonging. (Lesbian cohabiting woman, 25–34)

Twenty-one percent reported that complying increased negative feelings for their partner. Some of the feelings described were hate, resentment, and disgust, and some participants reported that they were less attracted to their partners. In some cases, the respondent had become disappointed in the partner because the gesture had not been reciprocated:

[It] caused me to believe my partner would take an equal share of responsibility and sacrifice in the future when they did not. (Less represented identity participant, 18–24)

Twenty percent reported that compliance had led to a better or stronger relationship*.* In some instances, the increased relationship satisfaction was described as a consequence of the partner being happy or satisfied:

My partner gets pleasure, and their well-being, of course, in its own way, has a positive effect on the relationship. (Heterosexual woman in relationship, 25–34)

On the other hand, fourteen percent reported that the relationship with their partner, in general, had suffered because of the compliance. In some cases, the decrease in relationship satisfaction was described as a secondary consequence:

If my desire doesn’t awake and my partner tries, then I’m sometimes frustrated, and if the relationship is already on shaky ground and sex “fails” then it reflects negatively on the relationship. (Lesbian woman in relationship, 25–34)

Seventeen percent described that the relationship had gotten worse by decreasing trust in the partner and the relationship, for instance:

Overall, I feel that compliance doesn’t necessarily affect the relationship if I accept it as part of the relationship and don’t talk about it to my partner. But then it affects me even more, when I feel I can’t trust my partner enough to talk to them about it. (Heterosexual woman in relationship, 25–34)

Thirteen percent reported that compliance had contributed to more distance in the relationship. Lastly, ten percent reported that compliance had resulted in them questioning the relationship.

Complying also made me think about the relationship in a new way; if I don’t feel sexually attracted to my partner, do I even want to be together? (Heterosexual single woman, 25–34)

#### Partner’s Response

The second overarching theme concerned how the partner responded to the situation. Thirty-four percent reported that a positive consequence of complying was that their partner was satisfied or happy. In some cases, this theme was presented together with the absence of something negative that not complying might have led to, indicating that this theme was at least to some extent related to avoidance motives for sex:

Fewer arguments about not having sex when my partner’s desires are satisfied. (Less represented identity participant, 25–34)

Twenty-two percent reported that their partner might get upset or hurt because of the compliance:

If my partner has noticed that I am not 100% on board, he has often taken offense and thought that I am doing it because I feel I have to. (Heterosexual single woman, 18–24)

On the other hand, 9% reported that their partner had become nicer or more understanding as a result of the respondent’s compliance.

#### Relationship Interaction

Some participants responded that compliance had affected the interaction with the partner. Twenty-six percent reported that compliance had led to conflicts with the partner. However, in some instances, it was unclear whether the conflicts were due to compliance or due to desire discrepancy in the relationship in general, for instance:

Friction over where to draw the line between pressure and suggestion. (Heterosexual married woman, 25–34)

While some participants reported that compliance had led to more tension and conflicts, 14% reported the opposite.

There has been a really non-existent amount of conflicts and disagreements about sex since we started complying. (Bisexual cohabiting woman, 35–44)

Twenty percent reported that complying to sex had contributed to creating a problematic sexual dynamic in the relationship:

It is more difficult for the partner to recognize sexual desire from sexual compliance. (Bisexual single woman, 25–34)

Ten percent reported that complying had affected their communication with their partner positively:

[It] has led to discussions about when and how to make the other person feel sexy and turned on. (Heterosexual cohabiting woman, 18–24)

While 10% reported that their communication had gotten better due to compliance, 9% reported the opposite.

Even if the situation has been resolved, the relationship has taken a negative turn in the sense that I haven’t felt completely safe telling them about my lack of desire because I’m afraid my partner will get angry. (Heterosexual single woman, 25–34)

Lastly, ten percent reported that complying had contributed to them avoiding their partner more.

#### Value Alignment

The last overarching theme concerned compliant behaviors aligning with values about intimate relationships held by the respondent. Twenty percent reported that compliance meant that they would have more sex, indicating that they value sex in the relationship. Some respondents expressed this value more explicitly:

I would rather have a relationship with lots of sex and not too little sex for me and my partner. (Bisexual woman in relationship, 25–34)

Twelve percent reported compliance as a way of maintaining the relationship:

In a long, close relationship, sexual compliance is one thing that makes a long relationship possible. (Heterosexual married woman, 45–54)

### Factors Contributing to Consequences

Lastly, the analysis yielded nine overarching themes for factors contributing to the consequences: communication, self-esteem, motives for sex, relationship factors, agency and self-knowledge, mental health and stress, psychological flexibility, societal norms, and past negative experiences.

#### Communication

Forty-nine percent reported that they felt that poor communication was one reason for them experiencing negative consequences of sexual compliance. In some instances, communication had been lacking:

Poor communication has really had a big effect; when partners haven’t been able to express how they feel about me telling them that I like them a lot but don’t want to have sex often or that for me, hugging/kissing/being close when my pants are still on is the best. (Less represented identity participant, 25–34)

In other instances, communication had not been constructive:

Difficulties communicating in the relationship (my own tone of voice easily becomes accusatory when talking about the subject, and for my partner, taking criticism has in general been difficult and even more difficult when talking about sex) […] (Heterosexual woman in relationship, 25–34)

In the same way that poor communication was the most frequently reported factor contributing to experiencing negative consequences, 47% perceived that good communication contributed to experiencing positive consequences.

Although the desires of both partners do not always coincide, good communication skills usually help to overcome situations. (Gay man in relationship, 25–34)

#### Self-Esteem

Thirty-six percent reported that they felt that issues with their self-esteem, self-confidence, or body image contributed to them experiencing negative consequences of compliance.

#### Motives for Sex

Some participants reported that the reason or motive they had for complying to sex contributed to the consequences. Twenty-three percent reported that they believed their tendency to please their partner or other people in general had contributed to the negative consequences:

On a personal level, excessive submission or the need to please has contributed to why I have complied and why I have become anxious afterward. (Heterosexual dating woman, 25–34)

On the other hand, 13% reported that they thought that either their enjoying pleasing the partner or their wanting their partner to be happy contributed to the positive consequences. In contrast with the people-pleasing tendencies, where the responses often highlighted that the individual felt a need for pleasing, these responses highlighted feeling personal joy from pleasing:

I have a strong desire to please and I want my partner to be happy. (Heterosexual cohabiting woman, 18–24)

Thirteen percent perceived that the positive consequences were due to them complying in order to achieve something positive (i.e., approach motives):

I know that if I agree to sex, 80% of the time I enjoy it too. (Heterosexual married woman, 25–34)

Similarly, twelve percent reported that they believed that avoidance motives for sex contributed to the negative consequences.

Desire to avoid conflict, don’t want to ruin the situation, complying seems easier than having a discussion. (Heterosexual single woman, 25–34)

#### Relationship Factors

Twenty-one percent reported that they felt that trust contributed to the positive consequences. Most of these responses were given using only keywords such as trust, trust in partner, and trust in relationship. Seventeen percent reported that they thought a good relationship, in general, contributed to the positively perceived consequences. Keywords used were, for instance, love, relationship satisfaction, and caring. On the other hand, 11% reported that a poor relationship in general or that issues in the relationship contributed to the negatively perceived consequences.

Twelve percent reported that shared views with their partner and a shared understanding of the situation contributed to the positive consequences.

A similar approach to sexuality. (Less represented identity participant, 35–44)

On the other hand, 6% reported that differing views between them and their partner contributed to the negative consequences.

Differing attitudes/beliefs about the meaning of sex and consequences of sex. (Less represented identity participant, 35–44)

Lastly, ten percent reported that the perceived negative consequences were a result of their partner being inconsiderate:

Partner ignored that I looked uncomfortable, lack of understanding from partner, pressure from partner. (Heterosexual woman in relationship, 18–24)

On the other hand, 8% reported that their partner being considerate contributed to the positively perceived consequences.

The empathy I have been able to get from my partner at certain moments. (Heterosexual single woman, 18–24)

#### Agency and Self-Knowledge

Nineteen percent reported that they believed that them not being assertive enough had contributed to the negative consequences:

And I have not always dared to interrupt the situation […] (Bisexual married woman, 35–44)

Sixteen percent reported that they thought a belief in themselves and their own agency contributed to the positive consequences.

I know I never really have to comply. (Heterosexual married man, 25–34)

Finally, whereas sixteen percent reported that the positive consequences were due to them knowing themselves (sexually) and their boundaries well, five percent reported the opposite; that their inexperience and lack of self-knowledge contributed to the negative consequences.

The ability to distinguish what is ok vs. what I don’t really want, that is, self-awareness. (Less represented identity participant, 25–34)

#### Mental Health and Stress

Twelve percent reported that their own personal issues might have contributed to the negative consequences:

Depression that was not a direct result of the relationship, but that also did not help with the situation. (Heterosexual single man, 25–34)

Mental health aspects covered common affective disorders and symptoms such as depression and anxiety, but also other difficulties such as feelings of shame related to sexuality. One participant also mentioned ADHD as a potential contributor. In some cases, the mentioned issues were linked to poor communication:

My mental illness was probably at the root of both my lack of sexual desire and my inability to communicate in a good, appropriate way with my partner about what I felt and wanted. (Bisexual single woman, 18–24)

Moreover, 6% reported stress or time issues as possible contributors to the negative consequences. For this category, most responses were short answers, such as “Little time together,” “Stress,” and “The rush of everyday life.”

#### Psychological Flexibility

Twelve percent reported that their open mindset contributed to the positive consequences. These descriptions included, for instance, an interest in sexuality and exploring different aspects of it, and understanding that there are several ways to be sexual with a partner, and being open to new sexual experiences:

Willingness to try new things. (Gay single man, 18–24).

Five percent reported that their capacity to get into sex or capacity for responsive desire contributed to the positive consequences.

Probably my capacity to get into it, when I have given sex a chance, even if I didn’t want it in the first place. (Heterosexual single woman, 25–34)

These descriptions also included being able to easily find pleasure and enjoy sex and were thus not strictly limited to desire.

#### Societal Norms

Ten percent reported that either societal norms or the participant’s own views or internalized norms on sexuality contributed to the perceived negative consequences. For instance, some responses highlighted the view of women’s desire and pleasure as secondary:

My own ideas, learned from a young age, of women’s desire and pleasure being secondary (“women just don't enjoy sex in the same way as men” and “it's normal for women not to orgasm during sex” and “sex is the same as male penetration and orgasm”), have contributed to a feeling that my own desire and pleasure in sex is secondary and that seeking my own pleasure causes feelings of guilt. (Heterosexual woman in relationship, 25–34)

Others noted society’s norms about having sex:

Thinking that we should have sex, more doing it because it is something we should than another better reason, that is, following the rules. (Heterosexual single woman, 25–34)

Some also noted negative or reserved attitudes toward sex:

Having a very sex-negative upbringing. (Gay single man, 18–24).

#### Past Negative Experiences

Lastly, 6% reported that they felt that past negative experiences or trauma contributed to the negative consequences. These included sexual and nonsexual trauma:

My own traumatic sexual experiences, such as being raped. (Heterosexual single woman, 25–34)

Decades of previous nonsexual trauma. (Less represented identity participant, 18–24)

Some answers were not explicitly referred to as traumatic but conveyed negative experiences such as feeling used or being disappointed.

### Possible Gender and Age Differences

Although most participants who provided free-text answers were cis women (*n* = 83), we did receive responses from 20 cis men, one transman, and four non-binary individuals. As the trans and non-binary individuals were very few, we did not examine their responses separately. However, we noticed that some of the more frequent categories among cis women were not reported by any cis men; for instance, negative feelings about self, decreased sexual desire, physical pain, negative feelings toward partner, and values sex in relationship.

Most themes were reported both among the 30 youngest and 30 oldest participants. Among the 30 youngest, no one reported the categories wasting time, time issues or stress, less tension/conflicts, partner behaving better, and capacity for responsive desire. Among the 30 oldest, no one reported negative feelings/thoughts about future, avoidance, worse communication, past negative experiences, and capacity for responsive desire.

## Discussion

We explored the perceived consequences of sexual compliance and possible factors contributing to these consequences by analyzing 107 free-text survey responses from mostly Finnish adults. We identified five overarching themes for personal consequences (emotions and mood, sexual experience, sexual desire, pressure and violations, and physical pain), four for relational consequences (relationship satisfaction, partner’s response, relationship interaction, and value alignment), and nine for possible contributors (communication, self-esteem, motives for sex, relationship factors, agency and self-knowledge, mental health and stress, psychological flexibility, societal norms, and past negative experiences).

### Consequences Consistent with Previous Compliance Literature

Some of the themes identified have been discussed previously in the context of sexual compliance. For instance, the most commonly reported personal theme, emotions and mood, corresponds to several of the negative aspects included in the previous studies on the consequences of compliance (Bay-Cheng & Bruns, 2016; Himanen & Gunst, 2023; O’Sullivan & Allgeier, 1998). While the previous studies have mainly emphasized negative emotions, some of our participants reported that sexual compliance increased their mood. Some responses suggested that at least some of these positive personal consequences may be mediated by relational aspects such as increased intimacy and connectedness with the partner.

In line with previous work (Vannier & O’Sullivan, 2010), several participants reported a decreased quality of sex. These reports concerned, for instance, a decreased amount of pleasure, sex becoming a routine and/or performance, and problems with relaxation and arousal. On the other hand, some participants also reported that sex turned out pleasurable, and many described that their desire awakened during sex. This is consistent with Basson’s (2000) theory of responsive desire, which has been used by clinical sexologists (e.g., Nagoski, 2015) and by couples themselves (e.g., Herbenick et al., 2014) as a motive for engaging in sex without feeling strong initial desire. Saying yes to (non-coercive) sexual activity can open the possibility of experiencing desire and pleasure. It is worth noting that our definition of sexual compliance specified the lack of desire to the beginning of the sexual activity. Our participants likely reported more positive consequences of compliance than they would have without this specification (i.e., because cases of responsive desire might then fall outside of the definition). However, it is possible that there was some confusion between the two concepts, as it may be difficult to pinpoint when exactly one has started to desire.

The pressure and violations theme aligns with previous studies on covert coercion (Conroy et al., 2015; Gavey, 1992). Some of our participants reported that societal norms about sex and relationships contributed to the negative consequences. Furthermore, while the partner may not have explicitly or implicitly pressured the respondent, sexual interdependence dilemmas (i.e., when one’s opportunities for sex with a partner are limited by the partner’s willingness, e.g., Day et al., 2015) can create situations in which one feels trapped because declining may have negative consequences for the relationship. Thus, it may be that some of the situations that are on a surface level free of pressure are not, in fact, completely free. It may also be that this type of pressure is more easily recognized in retrospect. Sexual consent is a complex and dynamic process, and in committed relationships, consent is often assumed unless the partner communicates their refusal (e.g., Willis & Jozkowski, 2019). Finally, although our compliance definition precluded pressure, some respondents may have misinterpreted this and described coercive situations.

Among the relational consequences, we found that some individuals reported that their partners behaved better as a result of sexual compliance. This finding is supported by previous daily diary research, showing that engaging in sexual activity predicts both the partner actually behaving more nicely or responsively and perceiving the partner as nicer (Dewitte et al., 2015). Together with the other positive relational consequences, our findings add to the literature suggesting that sexual compliance has a regulatory function in committed relationships (e.g., Muise & Impett, 2015; O’Sullivan & Allgeier, 1998).

### Consequences New to the Compliance Literature

We also identified several themes that have not received much attention in the compliance literature. For instance, some participants indicated that by complying, they were acting in accordance with their values about intimate relationships (e.g., wanting a relationship with frequent sex). The association between personally held values and societal norms remains unknown: It may be that at least some of these responses reflect internalized societal norms about frequent sex as important for being a good sexual partner. On the other hand, it is also plausible that sexual compliance is an expression of the individual’s agency to engage in activities that are considered meaningful, regardless of the lack of sexual desire.

Some participants also experienced physical pain. This is perhaps not surprising, as a decreased quality of sex was also reported as a negative consequence. A decreased quality of sex can affect the ability to become aroused and relaxed, which, in turn, makes the individual vulnerable to various forms of physical pain (e.g., Brauer et al., 2006). On the other hand, the pain literature also suggests that compliance is common among women who experience genital pain and that it is motivated by, for instance, not wanting to hurt the partner, guilt, and striving to affirm the image of being an ideal woman (Elmerstig et al., 2008, 2013). Thus, pain may be both a contributor to and a consequence of compliance.

Our findings also paint a more detailed picture of the potential negative relational consequences of sexual compliance. While previous research has highlighted the benefits of compliance for relationship satisfaction, we identified some negative consequences that may provide interesting targets for future research and clinical work. Such themes included, for instance, decreased trust in the partner, avoidant behavior, and vicious cycles of problematic sexual dynamics in which consent is assumed, and boundaries are gradually overstepped.

### Perceived Factors Contributing to Consequences

Communication was the most frequently reported contributor to both positive and negative consequences, highlighting communication as a central factor in addressing issues related to sexual compliance. Although the clinical literature on sexual desire discrepancy is sparse, at least one study of mitigation strategies for desire discrepancy (Vowels & Mark, 2020) indicated that the use of partnered strategies, including communication, was associated with higher levels of sexual and relationship satisfaction. In light of this, communication may promote positive consequences at least for relationship satisfaction and the quality of sex.

Motives for sex, identified as a possible contributor to both negative and positive consequences, has already received attention in the context of sexual compliance. Katz and Tirone (2009) linked avoidance motives with lower relationship satisfaction in compliant women, and Himanen and Gunst (2023) found that avoidance motives were associated with reporting more negative consequences, whereas approach motives were associated with fewer negative consequences. Because both studies were correlational, a possible causal relationship remains unknown; poor relationships may increase avoidant compliance.

Among the motives for sex theme, some participants reported that they were happy to please their partner. Being responsive to a partner’s sexual needs is also known as sexual communal motivation in the relationship research literature (Muise & Impett, 2015). Sexual communal motivation has previously been associated with several relationship benefits, such as partners being more satisfied and committed to the relationship. Thus, it is likely that sexual communal motivation contributes to particularly positive outcomes for the relational aspects, such as relationship satisfaction and the partner’s response.

Some identified themes (e.g., agency and self-knowledge, communication) loosely correspond to the work of Kennett et al. (2009) on sexual self-control, highlighting the link between compliance and strategies for managing unwanted sexual advances. We also found that mental health issues and lower self-esteem were perceived as contributing to the negative consequences. Although these factors have not yet been examined in the context of sexual compliance, studies linking personal factors such as lower sexual self-efficacy (Himanen & Gunst, 2023) and socioeconomic status (Bay-Cheng & Bruns, 2016) to negative consequences may at least partly explain this finding: Individuals with mental health issues and lower self-esteem might have fewer mental resources to deal with unwanted advances. Mental health problems might also affect sexual inhibition/excitation (Janssen & Bancroft, 2007), making it more difficult to become and remain aroused, and, in turn, to experience responsive desire. In addition, compliance may exacerbate negative feelings (e.g., anxiety and feeling inadequate) associated with various affective disorders. We thus predict that mental health problems and lower self-esteem may at least be relevant to the experience of negative emotions and mood and to a decreased quality of the sexual experience.

We also identified psychological flexibility among the perceived factors contributing to positive consequences. Some individuals reported having an open attitude toward sex and sexual experiences, indicating a flexible approach to sexual situations. Psychological flexibility has previously been highlighted as an important protective factor for psychological health (Kashdan & Rottenberg, 2010) and, more specifically, has been suggested as a possible strategy for coping with challenges related to sexual dysfunctions (Barsky et al., 2006; McCracken, 2013). The responses within the theme of psychological flexibility may also indicate that for some people, sexual compliance might not be seen as something that violates their personal needs and boundaries. In fact, we engage in activities we do not necessarily desire in various other areas of our lives (e.g., washing dishes or working out). It may be that for some people, sex is just one behavior among many.

Finally, our results suggest that sexual compliance may be harmful for individuals with past negative experiences, such as experiences of sexual abuse and assault. We speculate that individuals with a history of personal boundary violations may be particularly vulnerable to situations in which boundaries are crossed.

### Inter- and Intra-individual Differences in Consequences of Compliance

Our results indicated that the perceived consequences varied substantially across individuals; while the majority reported at least some negative consequences of compliance, approximately one-third reported either no or only positive consequences. The perceived consequences of compliance also varied substantially across individuals within specific domains. For instance, while some individuals perceived that compliance increased their self-esteem, others reported that it made them feel bad about themselves. Similarly, while some reported that compliance led to more conflict in the relationship, others reported that compliance made their relationships more harmonious. Although not analyzed separately, there were also instances where the perceived consequences varied within the same individual, such that compliance would sometimes make them feel better and sometimes make them feel worse. In summary, the inter-individual and intra-individual variance suggests that sexual compliance should be treated as a complex phenomenon, with outcomes depending on both the individual and the context rather than as a one-size-fits-all experience.

Although our results do not represent population-based estimates, our findings are consistent with previous frequencies from a large Finnish convenience sample (Himanen & Gunst, 2023), suggesting that it is more common to experience negative consequences than positive ones. In light of this, careful consideration is needed in cases of sexual compliance.

### Potential Gender and Age Differences

While the cis women in our sample reported all the categories, some categories were not reported by any of the cis men. It may be that some of these categories are more relevant to cis women; for example, physical pain may be more common among those who receive vaginal penetration (—although this may also be the case for anal penetration). However, it is worth noting that our sample of cis men was smaller than our sample of cis women, which may have also influenced whether a category was reported. Unfortunately, our study may also have missed aspects that are particularly relevant to gender minorities.

As with gender, most themes were reported in both age groups. While some categories were not reported by either the youngest or oldest individuals, we did not identify any clear age-related patterns. One possible difference was that the younger group did not report wasting time as a possible consequence, nor did they report time issues or stress as a possible contributor, suggesting that a stressful life may be more relevant in older age groups; many people in their 30 s and 40 s are juggling both parental responsibilities and more demanding jobs. However, both wasting time and time issues of stress were among the less frequently reported themes (both reported by 6% of the respondents). It is also worth noting that the age gap between the two age groups was relatively small.

Based on these preliminary findings, it appears that most of the themes are relevant across gender and age. While we found some differences, future quantitative studies should examine whether these trends are robust and whether individuals of different ages and genders interpret sexual compliance in the same way.

### Limitations and Future Directions

Some general limitations of the scope and nature of the data must be considered when interpreting our findings. As previously noted, most of our sample consisted of cis women, and the number of gender minority individuals, in particular, was small. To get a more complete understanding of the experiences of sexual compliance, future studies should aim to cover the full range of gender and sexuality intersections. Moreover, we focused on compliance within committed relationships. Although sexual compliance becomes particularly relevant in committed relationships due to relationship maintenance motives and sexual interdependence dilemmas, compliance is not exclusive to committed relationships (Willis et al., 2022). The experienced consequences of compliance outside committed relationships may differ from our findings. Due to small subgroup sizes, we did not compare the perceived consequences of compliance in different types of committed relationships (e.g., married vs. others where the stakes might be different). Besides, our query did not clarify if the perceived outcomes pertained exclusively to the current relationship. While being in a relationship was not an inclusion criterion, this could have led to varied interpretations among those in relationships. We only examined consequences with a positive or negative valence, though there may also be consequences that are considered neutral. However, we argue that valenced consequences are more relevant to the well-being than neutral consequences. In addition, our data were limited by the retrospective nature of the responses. Future research should address this limitation through prospective designs and intensive data collection.

About nine-tenths of our sample were Finnish and the rest were either North American or West European. There may be culture-specific themes that we did not manage to capture. It is worth noting that Finland ranks high on egalitarian gender-role attitudes (Kolpashnikova et al., 2020) and has long incorporated comprehensive sex education within its educational curriculum (Apter, 2011).

While we provided the participants with a written definition of sexual compliance and other key terms, there is still a risk that some might not have fully grasped the concept. Future in-depth qualitative research could delve into how people understand the concept. We saw no clear misunderstandings in responses, but occasionally it was hard to discern if something mentioned (e.g., “quarreling”) was viewed as a compliance consequence or related to low desire or desire discrepancy in general. Similarly, it was sometimes difficult to discern if the respondent perceived something they mentioned as a compliance consequence or just as part of their story. Consequences were also sometimes reported in a chain of events, indicating that—at least in the respondent’s experience—the consequence might be indirect. In such cases, co-authors inspected the first author’s interpretation. Because our study was hypothesis-generating, we were generally inclusive when there was no clear indication that the respondent misunderstood the question. However, future studies should establish these consequences of sexual compliance with precision, and in-depth interview studies should delve into how people understand and relate to the concept of sexual compliance.

Single-word responses such as “depression” were included in the analysis and frequency reports. All entries were reviewed by two independent coders, and any ambiguous responses were excluded. While we recognize the possibility of misinterpretation, we believe that excluding these responses would lead to the loss of valuable information, especially for categories where participants may have believed that elaboration was unnecessary. Nonetheless, we advise considering the reported frequencies with this context in mind.

Another limitation is that “poor/good communication” and “high/low self-confidence” were used as examples in the questions about potential factors contributing to the perceived consequences. We included these examples to make the question more concrete and to reduce misunderstandings. However, this may have increased the reports of communication and self-confidence. In fact, both examples were included in the responses, and communication was the most frequently mentioned contributing factor for both negative and positive consequences. Therefore, future studies need to examine its role as the most central factor. It is also worth noting that the reported frequencies illustrate the participants who mentioned a particular consequence or contributing factor unprompted. These percentages might be higher if the participants were presented with pre-selected themes.

Finally, there are slight variations in the definition of sexual compliance in the literature. While most scholars emphasize the lack of desire, phrasings often vary between unwanted sexual activity, sex one does not want or desire, and sexual activity without own desire (e.g., Khera et al., 2022; Vannier & O’Sullivan, 2010). It is unclear whether these phrases are interpreted similarly, for instance, in terms of aversion to the activity. Some scholars have also highlighted avoidance motives in the context of compliance (e.g., Impett & Peplau, 2003). Our definition of compliance as engaging in sexual activity “despite the lack (at least in the beginning) of sexual desire” is, as such, one of the broader ones, as it does not emphasize aversion and avoidance (and, moreover, specifies the lack of desire to the beginning of the event). Participants in our study likely reported more positive consequences than they would have if the definition had emphasized aversion and avoidance. In addition, our definition of sexual desire did not tie the desire to a specific sexual behavior. A person could arguably desire other types of activities than those being complied with. Consequently, our sample may reflect individuals whose lack of sexual desire is more generalized. Finally, some critique has been raised about the dichotomous conceptualization of sexual activity as either desired or undesired (Muehlenhard & Peterson, 2005). This conceptualization ignores an individual’s potentially ambiguous feelings and cognitions about sexual activity.

### Conclusions

We identified both themes that have been previously discussed in relation to sexual compliance and intimate relationships, as well as novel themes that may provide interesting targets for future research. We also noted considerable individual variability in perceived consequences, both in terms of experiencing any positive or negative consequences and in terms of whether compliance was perceived as improving or worsening specific domains of well-being. Our study lays the groundwork for future quantitative and longitudinal studies that examine causal relationships between sexual compliance and the identified themes.

### Supplementary Information

Below is the link to the electronic supplementary material.

Supplementary file1 (DOC 283 kb)

## Data Availability

The data that support the findings of this study are available on request from the corresponding author, Annika Gunst.
