# Supplementary material for: A Qualitative Content Analysis of Perceived Individual and Relational Consequences of Sexual Compliance and Their Contributors
Source: Arch Sex Behav. 2024 Jul 18;53(8):3025–41. doi: 10.1007/s10508-024-02948-9 (PMC11335786; doi:10.1007/s10508-024-02948-9)
Supplement: Supplementary file 1 — Supplementary file1 (DOC 283 kb) [file 10508_2024_2948_MOESM1_ESM.doc]

**Online Supplementary Material**

| Online Supplementary Table 1  *Perceived Personal and Relational Consequences of Sexual Compliance (n = 185)* | | |
| --- | --- | --- |
| Variable | *n* | % |
| Personal |  |  |
| Only negative consequences | 31 | 16.8 |
| Only positive consequences | 17 | 9.2 |
| Both negative and positive consequences | 73 | 39.5 |
| No consequences | 64 | 34.6 |
| Relational |  |  |
| Only negative consequences | 24 | 13.0 |
| Only positive consequences | 23 | 12.4 |
| Both negative and positive consequences | 75 | 40.5 |
| No consequences | 63 | 34.1 |
| Total |  |  |
| Only negative consequences | 23 | 12.4 |
| Only positive consequences | 18 | 9.7 |
| Both negative and positive consequences | 100 | 54.1 |
| No consequences | 44 | 23.8 |
| *Note.* Frequencies represent responses before recoding misplaced responses (described in the section Analytical Process). | | |

| Online Supplementary Table 2  *Demographic Characteristics Including Participants Who Perceived no Consequences of Sexual Compliance and Who Did Not Fill Out Free-text Responses on Sexual Compliance (n = 185)* | | |
| --- | --- | --- |
| Variable | n | % |
| Gender |  |  |
| Woman | 129 | 69.7 |
| Man | 50 | 27.0 |
| Transwoman | 1 | 0.5 |
| Transman | 1 | 0.5 |
| Non-binary | 4 | 2.2 |
| Sexual orientation |  |  |
| Heterosexual | 133 | 71.9 |
| Bisexual | 31 | 16.8 |
| Homosexual | 9 | 4.9 |
| Pansexual | 8 | 4.3 |
| Asexual | 4 | 2.2 |
| Relationship status |  |  |
| Single | 55 | 29.7 |
| In a dating relationship | 15 | 8.1 |
| In a committed relationship | 31 | 16.8 |
| In a consensual non-monogamous relationship | 9 | 4.9 |
| Cohabiting | 34 | 18.4 |
| Married | 39 | 21.1 |
| Other | 2 | 1.1 |
| Education |  |  |
| Middle/junior high school diploma (15 years) | 6 | 3.2 |
| Vocational school or high school diploma (18 years) | 38 | 20.5 |
| Bachelor’s degree (applied or university) | 75 | 40.5 |
| Master’s degree | 59 | 31.9 |
| Licentiate/doctorate degree | 6 | 3.2 |
| Other | 1 | 0.5 |
| Occupation |  |  |
| Studying | 45 | 24.3 |
| Employed or self-employed | 127 | 68.6 |
| Retired | 2 | 1.1 |
| Unemployed | 4 | 2.2 |
| Other | 7 | 3.8 |
| Monthly gross income |  |  |
| Less than 500€ | 17 | 9.2 |
| 500–999€ | 24 | 13.0 |
| 1,000–1,999€ | 28 | 15.1 |
| 2,000–2,999€ | 47 | 25.4 |
| 3,000–3,999€ | 37 | 20.0 |
| 4,000–4,999€ | 17 | 9.2 |
| 5,000–5,999€ | 5 | 2.7 |
| 6,000€ or more | 10 | 5.4 |
| Nationality |  |  |
| Finnish | 160 | 86.5 |
| West European | 7 | 3.8 |
| North American | 18 | 9.7 |
| Survey language |  |  |
| Finnish | 112 | 60.5 |
| English | 38 | 20.5 |
| Swedish | 35 | 18.9 |
| *Note.* Mean age = 31.12 years (*SD* = 9.57, range 18–63. Mean relationship duration for those in relationships (*n* = 128) = 91.92 months (*SD* = 74.93, range 14–370). | | |

| Online Supplementary Table 3  *Themes and Frequencies of Themes for the Six Research Questions* | | | | | | | | | |
| --- | --- | --- | --- | --- | --- | --- | --- | --- | --- |
| Research question | Theme | Freq. rank | Category | Unique freq. | | Total freq. | Cis men | Young | Old |
|  |  |  |  | *n* | % |  |  |  |  |
| Negative personal consequences (*n* = 83) | Emotion | 1 | Negative feelings^*^ | 40 | 48.2 | 75 | x | x | x |
|  | Emotion | 2 | Negative feelings about self^*^ | 28 | 33.7 | 54 |  | x | x |
|  | Pressure | 3 | Feeling violated/pressured | 20 | 24.1 | 28 | x | x | x |
|  | Experience | 4 | Decreased quality of sex | 18 | 21.7 | 27 | x | x | x |
|  | Pain | 5 | Physical pain | 17 | 20.5 | 19 |  | x | x |
|  | Desire | 6 | Decreased sexual desire^*^ | 15 | 18.1 | 18 |  | x | x |
|  | Pressure | 7 | Own boundaries unclear | 10 | 12.0 | 14 | x | x | x |
|  | Emotion | 8 | Negative feelings/thoughts about future | 7 | 8.4 | 10 |  | x |  |
|  | Experience | 9 | Wasting time | 5 | 6.0 | 6 |  |  | x |
| Positive personal consequences (*n* = 78) | Experience | 1 | Sex turned out good | 32 | 41.0 | 39 | x | x | x |
|  | Desire | 2 | Responsive sexual desire | 27 | 34.6 | 28 | x | x | x |
|  | Emotion | 3 | Positive feelings^*^ | 21 | 26.9 | 26 | x | x | x |
|  | Experience | 4 | Enjoy making partner happy | 16 | 20.5 | 16 | x | x | x |
|  | Experience | 5 | New experiences | 14 | 17.9 | 16 | x | x | x |
|  | Emotion | 6 | Positive feelings about self^*^ | 8 | 10.3 | 9 | x | x | x |
|  | Desire | 7 | Increased sexual desire^*^ | 4 | 5.1 | 4 | x | x | x |
| Negative relational consequences (*n* = 77) | Interaction | 1 | More tension/conflicts^*^ | 20 | 26.0 | 22 | x | x | x |
|  | Partner | 2 | Partner is hurt | 17 | 22.1 | 22 | x | x | x |
|  | Satisfaction | 3 | Negative feelings toward partner | 16 | 20.8 | 20 |  | x | x |
|  | Interaction | 4 | Problematic future sexual dynamic | 15 | 19.5 | 15 | x | x | x |
|  | Satisfaction | 5 | Decreased trust | 13 | 16.8 | 14 |  | x | x |
|  | Satisfaction | 6 | Worse relationship^*^ | 11 | 14.3 | 11 | x | x | x |
|  | Satisfaction | 7 | Distance in relationship | 10 | 13.0 | 13 |  | x | x |
|  | Satisfaction | 8 | Questioning relationship | 8 | 10.4 | 14 |  | x | x |
|  | Interaction | 9 | Avoidance | 8 | 10.4 | 8 |  | x |  |
|  | Interaction | 10 | Worse communication^*^ | 7 | 9.1 | 8 |  | x |  |
| Positive relational consequences (*n* = 81) | Satisfaction | 1 | Increased intimacy | 39 | 48.1 | 53 | x | x | x |
|  | Partner | 2 | Partner is satisfied | 28 | 34.6 | 35 | x | x | x |
|  | Value | 3 | Values sex in relationship | 16 | 19.8 | 21 |  | x | x |
|  | Satisfaction | 4 | Better relationship^*^ | 16 | 19.8 | 17 | x | x | x |
|  | Interaction | 5 | Less tension/conflicts^*^ | 11 | 13.6 | 11 | x |  | x |
|  | Value | 6 | Relationship maintenance | 10 | 12.3 | 10 | x | x | x |
|  | Interaction | 7 | Better communication^*^ | 8 | 9.9 | 8 |  | x | x |
|  | Partner | 8 | Partner behaving better | 7 | 8.6 | 7 |  |  | x |
| Factors contributing to negative consequences (*n* = 83) | Comm | 1 | Poor communication^*^ | 41 | 49.4 | 46 | x | x | x |
|  | Esteem | 2 | Low self-esteem | 30 | 36.1 | 39 | x | x | x |
|  | Motive | 3 | People-pleasing | 19 | 22.9 | 23 | x | x | x |
|  | Agency | 4 | Lack of assertiveness | 16 | 19.3 | 18 | x | x | x |
|  | Health | 5 | Own personal issues | 10 | 12.0 | 15 | x | x | x |
|  | Motive | 6 | Avoidance motives^*^ | 10 | 12.0 | 13 | x | x | x |
|  | Relation | 7 | Poor relationship^*^ | 9 | 10.8 | 11 |  | x | x |
|  | Relation | 8 | Inconsiderate partner^*^ | 8 | 9.6 | 12 |  | x | x |
|  | Norms | 8 | Norms and views on sexuality | 8 | 9.6 | 12 | x | x | x |
|  | Relation | 10 | Differing views and understanding^*^ | 5 | 6.0 | 9 | x | x | x |
|  | Health | 11 | Time issues or stress | 5 | 6.0 | 8 |  |  | x |
|  | NegExp | 12 | Past negative experiences | 5 | 6.0 | 6 |  | x |  |
|  | Agency | 13 | Inexperience and poor self-knowledge | 4 | 4.8 | 4 | x | x | x |
| Factors contributing to positive consequences (*n* = 76) | Comm | 1 | Good communication^*^ | 36 | 47.4 | 39 | x | x | x |
|  | Rela | 2 | Trust | 16 | 21.1 | 16 | x | x | x |
|  | Relation | 3 | Good relationship^*^ | 13 | 17.1 | 15 | x | x | x |
|  | Agency | 4 | Self-knowledge and boundaries | 12 | 15.8 | 15 |  | x | x |
|  | Agency | 5 | Belief in self and own agency | 12 | 15.8 | 14 | x | x | x |
|  | Motive | 6 | Sexual communal motivation | 10 | 13.2 | 12 | x | x | x |
|  | Motive | 7 | Approach motives^*^ | 10 | 13.2 | 10 | x | x | x |
|  | Flex | 8 | Openness | 9 | 11.8 | 11 | x | x | x |
|  | Relation | 9 | Shared views and understanding^*^ | 9 | 11.8 | 9 | x | x | x |
|  | Relation | 10 | Considerate partner^*^ | 6 | 7.9 | 6 |  | x | x |
|  | Flex | 11 | Capacity for responsive desire | 4 | 5.3 | 5 |  |  |  |
| *Note.* Themes with at least four unique responses are listed. Unique freq. = Unique individuals reporting the specific theme. Total freq. = Total number of codes for a specific theme, including duplicates from the same individual. ^*^ = Positive consequence/factor has mirrored negative version and vice versa. Cis men = Indicates whether category was reported among cis men (*n* = 20). All categories were reported by cis women. Young = Indicates whether category was reported among the 30 youngest individuals (aged 18–26 years) of those responding to at least one of the six questions. Old = Indicates whether category was reported among the 30 oldest individuals (aged 35–60 years) of those responding to at least one of the six questions. The *n*:s include individuals who, for instance, reported that they did not experience any negative personal consequences, but still mentioned a consequence among the negative relational consequences that ended up being treated as a negative personal consequence. Emotion = Emotions and mood, Pressure = Pressure and violations, Experience = Sexual experience, Pain = Physical pain, Desire = Sexual desire, Interaction = Relationship interaction, Partner = Partner’s response, Satisfaction = Relationship satisfaction, Value = Value alignment, Comm = Communication, Esteem = Self-esteem, Motive = Motives for sex, Agency = Agency and self-knowledge, Health = Mental health and stress, Relation = Relationship-related, Norms = Societal norms, , Flex = Psychological flexibility, NegExp = Past negative experiences. | | | | | | | | | |

| Online Supplementary Table 4  *Illustrative Examples of Categories* | | | |
| --- | --- | --- | --- |
| Research question | Theme | Category | Illustrative Example |
| Negative personal consequences | Emotions and mood | Negative feelings | “Negative consequences have included, for example, anxiety that has risen during the situation. In the situation, it has still felt like I just have to get over the anxiety. I have also felt anxious afterward, resulting in tearfulness.” |
|  |  |  | “I sometimes cannot escape the thought patterns and negative emotions associated with sexual compliance; they come up randomly sometimes when I try to sleep at night or when I have consensual and desired sex with other people.” |
|  |  | Negative feelings about self | “In the worst case: lack of self-respect and a feeling of having done violence to oneself.” |
|  |  |  | “Feeling worthlessness, shame of saying yes when you might not want to.” |
|  |  | Negative feelings/thoughts about future | “Compliance also increased the fear that this was to be expected in the future and contributed to anxiety when going to bed, knowing that the other person would be interested in having sex and I might not be.” |
|  |  |  | “I am scared to enter another relationship because of the obligations I feel to have sex with that person and the fear of enduring frequent sexual compliance again.” |
|  |  |  | “Fear of never being able to enjoy sex again.” |
|  | Pressure and violations | Feeling violated/pressured | “I have felt obliged to say yes and felt guilty if I refused completely.” |
|  |  |  | “The societal pressure to be sexy in a relationship is ever-present.” |
|  |  |  | “Sometimes I feel pressure to pretend to be more interested than I am at the moment so as not to offend my partner.” |
|  |  | Own boundaries unclear | “Being flexible feels difficult. Am I overstepping my own boundaries? Am I allowed to just not want to? Do I need to right now?” |
|  |  |  | “Constant shifting of boundaries (after I agree to do X even though I don’t want to, I might as well agree to do Y even though I really don’t want to, etc.)” |
|  | Sexual experience | Decreased quality of sex | “I switch off completely and don’t feel pleasure in my body; for instance, when my partner wants to please me and gives me oral sex, I’m just bored and pretend to enjoy it.” |
|  |  |  | “The missus wanted a child so badly that sex was just a performance.” |
|  |  |  | “Having sex becomes something you do routinely rather than something you really want to do.” |
|  |  |  | “…if you have sex for someone else, it […] is quite tense.” |
|  |  |  | “Sometimes we stopped having sex because we both realized that sex doesn’t work with half an erection.” |
|  |  | Wasting time | I don’t just let myself be or rest, even if it would be beneficial for me. I may be tired, especially in the evenings when I have sex, or I may not have slept enough in the morning because I had sex.” |
|  |  |  | “I may leave some things undone or put them off, for example, housework.” |
|  | Physical pain | Physical pain | “Physical pain (after penetrative sex when I was not relaxed enough).” |
|  |  |  | “Sex causes more pain if I am not properly aroused. Sometimes there may be pre-existing sores or other pain or irritation in the genitals that are forgotten when I am aroused but return worse after sex.” |
|  |  |  | “Vaginismus - I’m currently in my first ever non-compliant intimate relationship but find vaginal penetration impossible.” |
|  | Sexual desire | Decreased sexual desire | “A long-term consequence is a decrease in sexual desire.” |
|  |  |  | “A pronounced experience of lack of sexual desire.” |
| Positive personal consequence | Sexual experience | Sex turned out good | “Sex has always been good, even if it has been compliant, and it has made me feel good.”  “Sexual pleasure could sometimes make the interaction worth it.” |
|  |  |  | “It has resulted in sexual pleasure and enjoyment. By saying yes, even if you don’t want to at that moment, you usually gain more than you lose.” |
|  |  | Enjoy making partner happy | “I feel good when I see that I can give pleasure to my partner.” |
|  |  |  | “I know that sex always makes my partner happy, so I’m happy to do it even if it’s not always fun in the first place.”  “It feels good to consider the other [person].” |
|  |  | New experiences | “I’ve found new things that arouse me, although I was a bit skeptical at first (okay then, let’s try it if you want).” |
|  |  |  | “As a result, I have a much more relaxed attitude to sex.” |
|  |  |  | “Bad experiences teach. Not everything has to be tolerated. And you learn to recognize assholes.” |
|  | Sexual desire | Responsive sexual desire | “So, in a way, the desire is there between your ears all the time, but sometimes it’s difficult to arouse it physically, especially in a long relationship. In such a situation, I know that with my current partner, I’m very likely to become aroused and find desire through sexual activity.” |
|  |  |  | “[Compliance] can help to awaken my own desire; I have always ended up enjoying sex—a bit like testing the ice with a stick to see if the desire will ignite.” |
|  |  | Increased sexual desire | “It has increased my own desire, often already during the act itself but also in general.” |
|  |  |  | “I have a much more relaxed attitude towards sex and as a result, I even have a bit more desire myself.”  “An increase in my own sexual desire in the moment and also afterward. A couple of consecutive times [it] has raised my low desire for a longer period of time.” |
|  | Emotions and mood | Positive feelings | “Made me happy in the moment.” |
|  |  | Positive feelings about self | “My self-esteem is boosted by the fact that my partner finds me beautiful, sexy, and desirable.” |
| Negative relational consequences | Relationship satisfaction | Worse relationship | “If my desire doesn’t awake and my partner tries, then I’m sometimes frustrated, and if the relationship is already on shaky ground and sex “fails” then it reflects negatively on the relationship.” |
|  |  |  | “It was humiliating for him and affected his self-esteem and, therefore, our whole relationship.” |
|  |  | Negative feelings toward partner | “[It] caused me to believe my partner would take an equal share of responsibility and sacrifice in the future when they did not.” |
|  |  |  | “I have given pleasure to another person, but they have not given pleasure to me. And it hasn’t even occurred to them that they could or should take me into consideration.” |
|  |  | Decreased trust | “Overall, I feel that compliance doesn’t necessarily affect the relationship if I accept it as part of the relationship and don’t talk about it to my partner. But then it affects me even more, when I feel I can’t trust my partner enough to talk to them about it.” |
|  |  |  | “This has led to […] a slight lack of trust in the relationship, but only for a short time.” |
|  |  | Questioning relationship | “A feeling difficult to define that the relationship is not working from both sides.” |
|  |  |  | “Complying also made me think about the relationship in a new way; if I don’t feel sexually attracted to my partner, do I even want to be together?” |
|  |  | Distance in relationship | “Distance between me and them caused by avoidance. The relationship becoming distant.” |
|  |  |  | “[A] wish to have more space between me and my partner.” |
|  | Partner’s response | Partner is hurt | “If my partner has noticed that I am not 100% on board, he has often taken offense and thought that I am doing it because I feel I have to.” |
|  |  |  | “My rather passive compliance did not make him happy in the long term even though it satisfied him physically. He still didn’t feel desired and in the long run (a few years later) and made him question his manhood and desirability.” |
|  | Relationship interaction | Problematic future sexual dynamic | “It is more difficult for the partner to recognize sexual desire from sexual compliance.” |
|  |  |  | “As sex in the last few years was often dependent on one of us complying, a kind of debt spiral developed in which both felt obliged to say yes because the other had said yes the last time.” |
|  |  | More tension/conflicts | “Arguing as a result of being frustrated.” |
|  |  |  | “Friction over where to draw the line between pressure and suggestion.” |
|  |  | Avoidance | “For a while it felt okay to comply, but then I gradually started avoiding sex more and more, to the point that it completely disappeared from our relationship, which later ended for various reasons.” |
|  |  |  | “You also start to behave, perhaps consciously or unconsciously, so as not to behave in a seductive way or in a way that would promote sex.” |
|  |  | Worse communication | “Even if the situation has been resolved, the relationship has taken a negative turn in the sense that I haven’t felt completely safe telling them about my lack of desire because I’m afraid my partner will get angry.” |
|  |  |  | “Lack of communication, misunderstandings […]” |
| Positive relational consequences | Relationship satisfaction | Increased intimacy | “The sexual connection is maintained and sometimes strengthened, positive sexual experiences reinforce a sense of intimacy and belonging.” |
|  |  |  | “When we have sex, I feel connected and close to him in a pure and deep way that can only be accessed sexually.” |
|  |  | Better relationship | “It really strengthens us as a couple when we can agree on things and come to a mutual understanding.” |
|  |  |  | “My partner gets pleasure, and their well-being, of course, in its own way, has a positive effect on the relationship.” |
|  | Partner’s response | Partner is satisfied | “My man feels better and is in a better mood when he gets cuddles, strokes, and orgasms.” |
|  |  |  | “Fewer arguments about not having sex when my partner’s desires are satisfied.” |
|  |  |  | “It kept him satisfied so he would not break up with me.” |
|  |  | Partner behaves better | “Sometimes if my partner gets frustrated, then after sex it’s in general easier to be around them.” |
|  |  |  | “My partner is happier, which makes it nicer to be around him.” |
|  | Value alignment | Values sex in relationship | “A steady amount of sex.” |
|  |  |  | “I would rather have a relationship with lots of sex and not too little sex for me and my partner.” |
|  |  | Relationship maintenance | “In a long, close relationship, sexual compliance is one thing that makes a long relationship possible.” |
|  |  |  | “A compromise that has kept us together so far.” |
|  | Relationship interaction | Less tension/conflicts | “There has been a really non-existent amount of conflicts and disagreements about sex since we started complying.” |
|  |  |  | “If the amount of sex decreases, we start arguing more.” |
|  |  | Better communication | “Good discussions on the subject which also led to other deeper discussions.” |
|  |  |  | “[It] has led to discussions about when and how to make the other person feel sexy and turned on.” |
| Factors contributing to negative consequences | Communication | Poor communication | “Poor communication has really had a big effect; when partners haven’t been able to express how they feel about me telling them that I like them a lot but don’t want to have sex often or that for me, hugging/kissing/being close when my pants are still on is the best.” |
|  |  |  | “Not discussing the physical aspects of the relationship, especially sex.” |
|  |  |  | “Difficulties communicating in the relationship (my own tone of voice easily becomes accusatory when talking about the subject, and for my partner, taking criticism has in general been difficult and even more difficult when talking about sex) […]” |
|  |  |  | “Talking about sex has been difficult, and we have blamed each other.” |
|  | Self-esteem | Low self-esteem | “My low self-confidence, believing that I’m worthless and inadequate […]” |
|  |  |  | “My own low self-esteem has a negative impact on everything. I don’t like to be touched because I find my body really disgusting and as a result the amount of sex has decreased a lot. My partner has never said or done anything that would have led to low self-esteem, quite the opposite, but in my own head it's really hard to change my attitudes toward my own body, no matter who says what.” |
|  | Motives for sex | People-pleasing | “That I’m a people pleaser […]” |
|  |  |  | “On a personal level, excessive submission or the need to please has contributed to why I have complied and why I have become anxious afterward.” |
|  |  | Avoidance motives | “Desire to avoid conflict, don’t want to ruin the situation, complying seems easier than having a discussion.” |
|  |  |  | “I’m also afraid that my spouse will think that I don’t think they are sexy or desirable.” |
|  | Agency and self-knowledge | Lack of assertiveness | “And I have not always dared to interrupt the situation […]” |
|  |  |  | “[An] inability to set boundaries and stand up for myself.” |
|  |  | Inexperience and poor self-knowledge | “Being new to relationships” |
|  |  |  | “Poor perception of my own needs” |
|  | Mental health and stress | Own personal issues | “Anxiety tendency” |
|  |  |  | “Depression that was not a direct result of the relationship, but that also did not help with the situation.” |
|  |  |  | “My mental illness was probably at the root of both my lack of sexual desire and my inability to communicate in a good, appropriate way with my partner about what I felt and wanted.” |
|  |  | Time issues or stress | “Little time together” |
|  |  |  | “Stress” |
|  |  |  | “The rush of everyday life.” |
|  | Relationship-related | Inconsiderate partner | “My partner believed that everyone around them was the problem, never themselves, and internalized ableism that they could never be stupid or crazy, or wrong.” |
|  |  |  | “Partner ignored that I looked uncomfortable, lack of understanding from partner, pressure from partner.” |
|  |  | Poor relationship | “In a previous relationship, the relationship was simply so bad to begin with that it only made it worse.” |
|  |  |  | “Too little intimacy in the relationship.” |
|  |  | Differing views and understanding | “Differing attitudes/beliefs about the meaning of sex and consequences of sex.” |
|  |  |  | “Too different lifestyles, values and visions for the future only added to our difficulties.” |
|  | Societal norms | Norms and views on sexuality | “My own ideas, learned from a young age, of women’s desire and pleasure being secondary (“women just don't enjoy sex in the same way as men” and “it's normal for women not to orgasm during sex” and “sex is the same as male penetration and orgasm”), have contributed to a feeling that my own desire and pleasure in sex is secondary and that seeking my own pleasure causes feelings of guilt.” |
|  |  |  | “Getting stuck in old ideas about women (A woman is a giver, not a taker)” |
|  |  |  | “Thinking that we should have sex, more doing it because it is something we should than another better reason, that is, following the rules.” |
|  |  |  | “Having a very sex-negative upbringing.” |
|  | Past negative experiences | Past negative experiences | “Decades of previous nonsexual trauma.” |
|  |  |  | “My own traumatic sexual experiences, such as being raped.” |
|  |  |  | “Previous experience of sexual abuse and grooming (as a child).” |
| Factors contributing to positive consequences (*n* = 76) | Communication | Good communication | “We communicate often how we are feeling and that we may need to stop but we want to try. Without that communication there would probably be more pressure and make things more uncomfortable.” |
|  |  |  | “Good communication and trust have allowed me to say that I don’t always feel desire right away but that my partner is welcome to initiate sex and exhibit sexual behavior because I’ll probably latch on pretty quickly.”  “Although the desires of both partners do not always coincide, good communication skills usually help to overcome situations.” |
|  | Relationship-related | Trust | “I can trust that if I really want to stop, my partner is ready to stop at any time.” |
|  |  | Good relationship | “My current relationship is very stable.” |
|  |  |  | “Basically feeling good and safe with my partner.” |
|  |  | Shared views and understanding | “A similar approach to sexuality.” |
|  |  |  | “A shared understanding of sexual compliance.” |
|  |  | Considerate partner | “The fact that my partner values my opinion is also important in the relationship.” |
|  |  |  | “The empathy I have been able to get from my partner at certain moments.” |
|  | Agency and self-knowledge | Belief in self and own agency | “I experience sexual consent as my choice and my investment in the relationship, rather than feeling that I have no choice in the matter.” |
|  |  |  | “The experience of autonomy and independence (that even if I’m in a relationship, I’m in it because I want to be with that person, not because I can’t stand being alone or can’t get by on my own).”  “I know I never really have to comply.” |
|  |  | Self-knowledge and boundaries | “The ability to distinguish what is ok vs. what I don’t really want, that is, self-awareness.” |
|  |  |  | “The fact that I know what I like and what arouses me.” |
|  | Motives for sex | Approach motives | “I know that my own desire usually kicks in eventually.” |
|  |  |  | “I know that if I agree to sex, 80% of the time I enjoy it too.” |
|  |  | Sexual communal motivation | “I have a strong desire to please and I want my partner to be happy.” |
|  |  |  | “Caring about the other person and their needs.” |
|  | Psychological flexibility | Openness | “Willingness to try new things.” |
|  |  |  | “An interest in sexuality and learning about my own sexuality since I was a child.” |
|  |  | Capacity for responsive desire | “Probably my capacity to get into it, when I have given sex a chance, even if I didn’t want it in the first place.” |
|  |  |  | “My ability to enjoy sex.” |
